# A seroepidemiological survey of adenovirus type 7 circulation among healthy adults in China and in Sierra Leone, West Africa

**DOI:** 10.3389/fpubh.2023.1095343

**Published:** 2023-02-06

**Authors:** Busen Wang, Jianhua Li, Shipo Wu, Yudong Wang, Yi Chen, Yanfang Zhai, Xiaohong Song, Zhenghao Zhao, Zhe Zhang, Jinlong Zhang, Rui Yu, Lihua Hou, Wei Chen

**Affiliations:** ^1^Vaccine and Antibody Engineering Laboratory, Beijing Institute of Biotechnology, Beijing, China; ^2^Zhejiang Provincial Center of Disease Control and Prevention, Hangzhou, China

**Keywords:** HAdV7, neutralization assay, antibodies, epidemiology, seroprevalence

## Abstract

Adenovirus type 7 (HAdV7) is one of the most pathogenic human adenoviruses (HAdVs) and can cause severe illness and even death, particularly in people with weakened immune systems. Many countries worldwide have experienced epidemics of this highly contagious pathogen, including China and Sierra Leone; however, studies describing the seroprevalence of anti-HAdV7 neutralizing antibodies (nAbs) are still lacking. Herein, we established an efficient neutralization assay based on a recombinant luciferase-expressing HAdV7 virus (HAd7-Luc) to monitor historical HAdV7 infections and predict outbreak distributions. Among the 2,350 serum samples collected from eight sites in China and Sierra Leone in this cross-sectional serological survey, the overall proportion of anti-HAdV7-seropositive individuals was nearly 60%, with higher seroprevalence rates in Sierra Leone than in China. Regionally, HAdV7 nAb titers were higher in China than in Sierra Leone and showed a geographic variation across different regions. Regardless of the location, the seropositive rate of HAdV7 nAb was lower than that of HAdV5 nAb, as was the nAb titer. The prevalence rates of antibodies against HAdV7 and HAdV5 were both related to age but not to sex. In addition, serologic cross-reactions were rarely observed among people infected with HAdV7 and HAdV5. These results indicate a humoral immune response acquired through endemic HAdV7 infection and enrich the understanding of not only the epidemiological prevention and control of HAdV7 but also the clinical application of HAdV7-based vaccines or gene therapy tools.

## Introduction

Human adenoviruses, comprising seven species (HAdV-A to G) and more than 100 assigned types (1), have occurred epidemically in both developing and developed regions in recent years. Different type induces distinct clinical symptoms. For instance, respiratory infections have been associated mostly with HAdV-B type (HAdV-3,−7,−21,−55), HAdV-C type (HAdV-1,−2,−5,−6) and HAdV-E (HAdV-4) type (2–5). Among these types, HAdV7 from subfamily B is one of the most pathogenic HAdV type and causes a broad spectrum of clinical symptoms, including fever, bronchitis, pneumonia, gastroenteritis, respiratory failure and even death (6–8); HAdV7 usually spreads by direct or indirect personal contact. The pathogenesis of HAdV7 infection results from the combined effect of multiple factors. Newborns, children, elderly individuals or immunocompromised persons are more susceptible to HAdV7 infection than their counterparts, and external factors such as overcrowding, a closed environment, and a large population contribute to HAdV7 transmission. In addition, HAdV7 infection cooccurring with respiratory syncytial virus or rhinovirus can produce outbreaks of severe pneumonia (9) and even increase the case-fatality rate of measles (10).

HAdV7 infection is fairly common among populations. During the last half century, numerous HAdV7 epidemics have occurred worldwide, including in China (6, 9, 11–14), Korea (15), Vietnam (10), Malaysia (16), the Philippines (17), Brazil (4), and the Americas (3, 18), especially in densely populated communities, such as schools (19), hospital wards (20), chronic care facilities (21, 22), and military training camps (23). The data in China are just starting to be numerous but still rare in Sierra Leone. Besides, few epidemiologic studies have described the prevalence of naturally occurring neutralizing antibodies (nAbs) against HAdV7 among populations in the two countries. In addition, effective adenovirus vaccines for the general public and specific treatments for HAdV7 infection are currently unavailable, although a prophylactic vaccine against adenovirus type 4 and 7 has been approved by the U.S. Food and Drug Administration for use in only military personnel (24, 25). Therefore, serological tests for HAdV7 infection are of great value in understanding the epidemic situation, controlling HAdV7 dissemination and preventing outbreaks.

In the present study, we established a rapid and reproducible neutralization assay using a newly constructed recombinant luciferase-expressing HAdV7 virus (HAdV7-Luc) to describe the epidemiology of HAdV7 infection in China and West Africa. In addition, we performed comprehensive analyses of the potential associations of age and sex with HAdV7 nAb seroprevalence, as well as serological cross-reactivity between HAdV7 and HAdV5, which has been comparatively developed and widely used for protein delivery in practices such as gene therapy and vaccine development, to establish an endemic surveillance network for HAdV7 infection and future development of superior alternative HAdV-based vectors in vaccine development.

## Results

### Optimization of the conditions of the HAdV7-Luc-based neutralization assay

**Figure 1:**
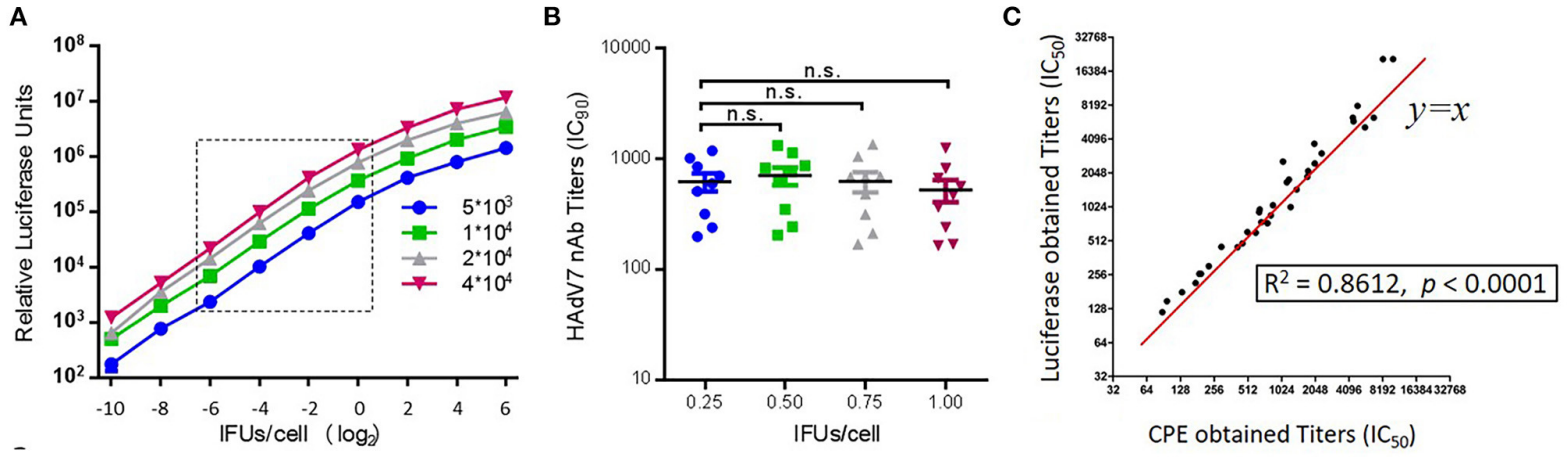
Establishment of an HAdV7-Luc-based neutralization assay. **(A)** HAdV7-Luc recombinant virus was serially diluted and added to different quantities of A549 cells in a 96-well plate, and luciferase was measured at 24 h post-infection. **(B)** Ten HAdV7-positive serum samples were incubated with HAdV7-Luc at four fixed IFU/cell ratios (0.25, 0.50, 0.75, and 1.00). After the neutralization assay was performed, serum titers were calculated the dilution level at which 90% of luciferase expression was inhibited. The difference between groups was analyzed by one-way ANOVA. **(C)** HAdV7-positive serum samples were tested by two different neutralization assays, and the IC_50_ values obtained by the luciferase expression inhibition assay (y-axis) and the CPE assay (x-axis) were compared and analyzed by Spearman's correlation coefficient.

A specific HAdV7-Luc recombinant virus was constructed by replacing the E1A region with a luciferase expression cassette and used for the neutralization assay. To determine the optimal conditions for HAdV7-Luc infection of target A549 cells, infections with HAdV7-Luc were performed using different concentrations of viral particles. A linear relationship between the fluorescence value and multiplicity of infection (MOI) was obtained at MOI = 0.0625–1 (Figure 1A), and no significant difference was observed when using different infectious doses (MOI = 0.25, 0.50, 0.75, or 1.00) to detect the nAb values of ten HAdV7-positive serum samples (one-way ANOVA test, *n* = 10, *p* > 0.05) (Figure 1B). Therefore, an infectious dose with a MOI value of 0.75 was chosen as the optimal viral dose for this assay. To further verify the reliability of the assay, we measured the same serum samples using the traditional CPE (cytopathic effect) method, and the results showed good consistency (*R*^2^ = 0.8612, *p* < 0.0001) (Figure 1C). These results demonstrated the stability and repeatability of the HAdV7-Luc-based neutralization assay.

### Seroprevalence of HAdV7 neutralizing antibodies in the general populations of China and Sierra Leone

**Figure 2:**
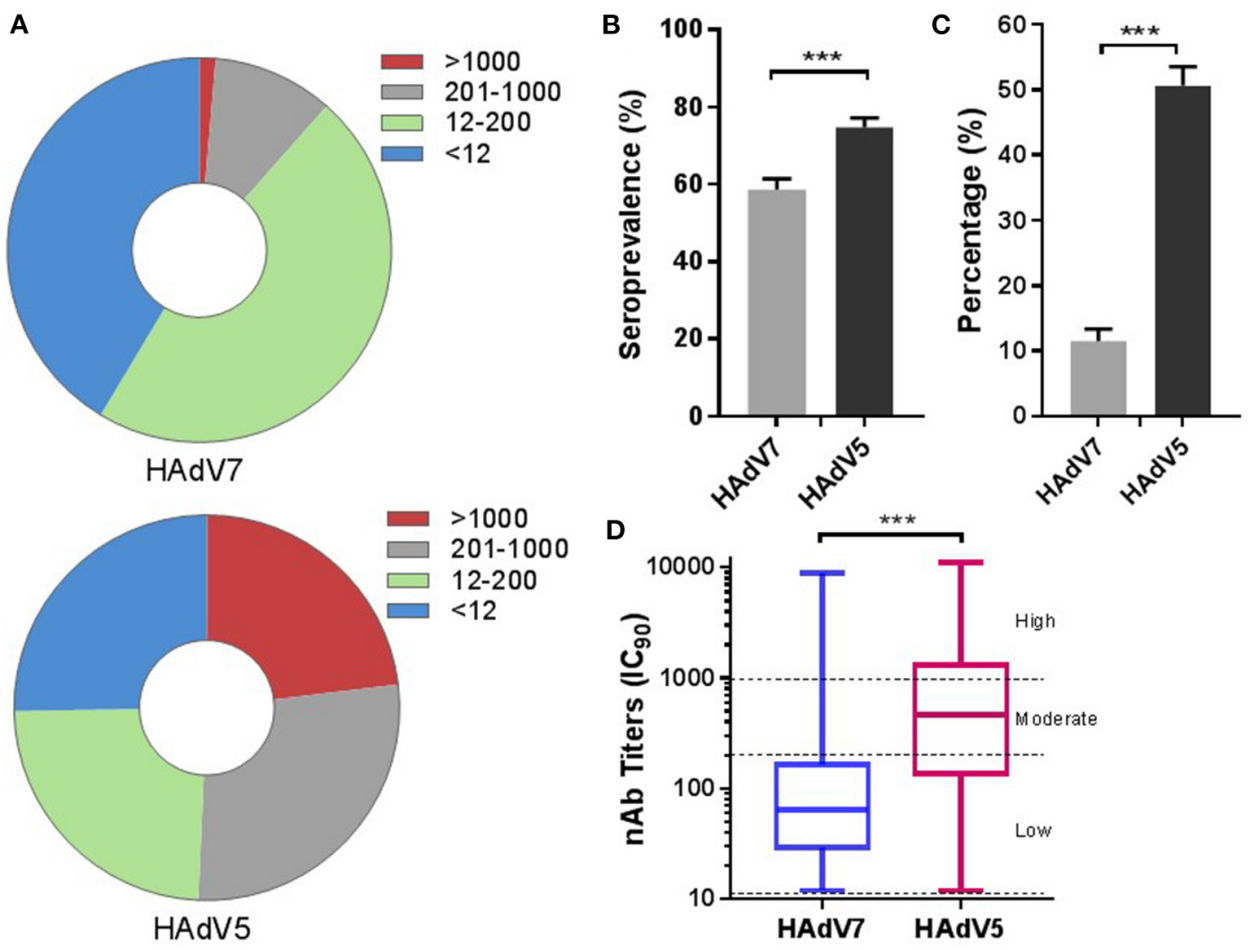
The overall seroprevalences of HAdV7 and HAdV5 nAbs (*n* = 2,350). The nAb titers of HAdV7 and HAdV5 were tested using HAdV7-Luc- and HAdV5-Luc-based neutralization assays (IC_90_), respectively. The nAb distributions **(A)** and seropositive rates **(B)** of HAdV7 and HAdV5 are shown. The data were analyzed by the Pearson chi-square test. **(C)** The percentages of serum samples with moderate or high levels of HAdV7 and HAdV5 nAbs are shown. The comparison was performed with the Pearson chi-square test. **(D)** The nAb titers of HAdV7- and HAdV5-positive serum samples analyzed by the Mann–Whitney test. n.s., nonsignificant, *p* > 0.05; *p, between 0.01 and 0.05; **p, between 0.01 and 0.001; ***p < 0.001.

We first evaluated the overall prevalence of nAbs against HAd7-Luc in sera collected from 2,350 participants compared with that of nAbs against HAd5-Luc. HAdV nAb titers were divided into four subgroups (< 12, negative; 12–200, low; 200–1,000, moderate; >1,000, high), similar to those reported in other HAdV seroprevalence studies (26). Intriguingly, most infected people had low nAb titers (47.1%; 95% CI: 45.0–49.1%) against HAdV7, while approximately even distribution of acquired nAb titers were observed for HAdV5 (Figure 2A). The overall seroprevalence of anti-HAdV was higher for HAdV5 (74.7%; 95% CI: 73.0–76.5%) than for HAdV7 (58.6%; 95% CI: 56.6–60.6%) (Pearson chi-squareare test, *p* < 0.001) (Figure 2B). It was found that 11.5% (95% CI: 10.2–12.8%) of the participants had moderate or high HAdV7 nAb titers (>200), and 1.4% (95% CI: 0.9–1.8%) had very high titers (>1,000); these proportions were much lower than the corresponding rates of 50.7% (95% CI: 48.7–52.7%) and 23.1% (95% CI: 21.4–24.8%) of participants with HAdV5 nAb titers (Pearson chi-squareare test, *p* < 0.001) (Figure 2C). The overall geometric mean titer of HAdV7 nAb among HAdV7-seropositive samples was 74.4 (95% CI: 69.9–79.3), which was much lower than that for HAdV5 (408.0; 95% CI: 379.0–439.3) (Mann–Whitney test, *p* < 0.001) (Figure 2D). These results suggested that nAbs against HAdV7 were less prevalent and were present at lower titers than nAbs against HAdV5.

**Figure 3:**
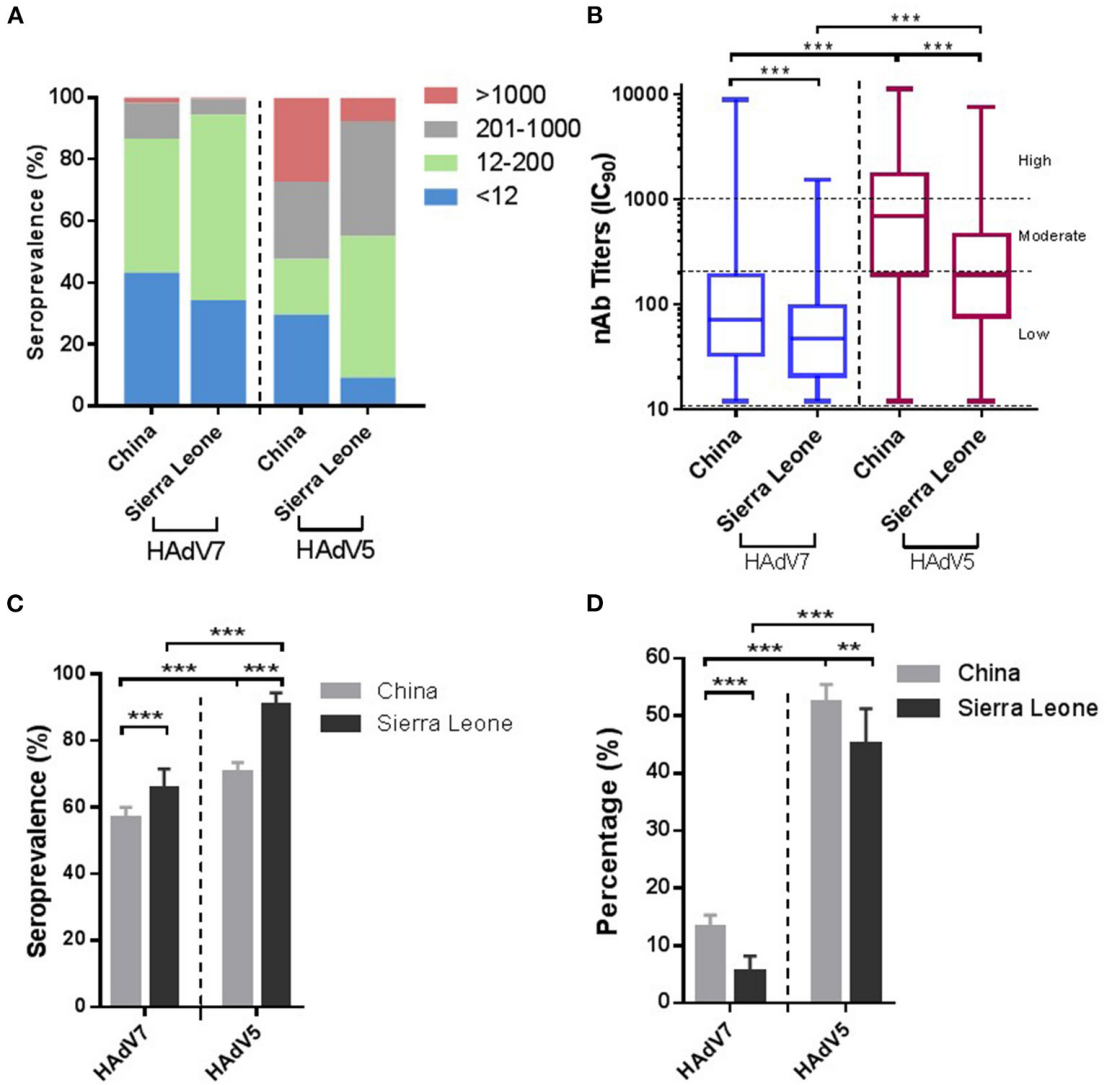
Seroprevalences of HAdV7 and HAdV5 nAbs in China and Sierra Leone. **(A)** The nAb distributions in China and Sierra Leone. **(B)** The nAb titers of HAdV7- and HAdV5-positive serum samples in China and Sierra Leone, analyzed by the Mann–Whitney test. The seropositive rates **(C)** and percentages of serum samples with moderate or high levels of nAbs **(D)** in China and Sierra Leone were analyzed by the Pearson chi-square test. n.s., nonsignificant, *p* > 0.05; *p, between 0.01 and 0.05; **p, between 0.01 and 0.001; ***p < 0.001.

We further analyzed the samples collected from China and Sierra Leone respectively. As shown in Figure 3, the samples from Sierra Leone had higher seroprevalence rates (65.7%; 95% CI: 61.5–69.8%) but lower nAb titers (49.7; 95% CI: 44.6–55.3) and the proportion (5.4%; 95% CI: 3.4–7.4%) of HAdV7 nAbs at a moderate to high level (>200) were lower than the corresponding values of 13.2% (95% CI: 11.6–14.7%) in China (*p* < 0.001) (Figures 3A–D). Besides, among all regions and adenovirus types, participants from Xizang and Gansu had the highest proportions of high preexisting titers (>1,000) (Supplementary Figure S1A). For HAdV7, the highest seroprevalence and nAb titers were observed in Xizang (Supplementary Figures S1B, C). In addition, the proportion of moderate to high nAb titers (>200) appeared to be relatively higher in Xizang, Xinjiang, and Beijing (Supplementary Figure S1D). These results indicated that there were geographic variations in the prevalence of natural HAdV7 infection.

### Age-related increase in HAdV7 nAbs

**Figure 4:**
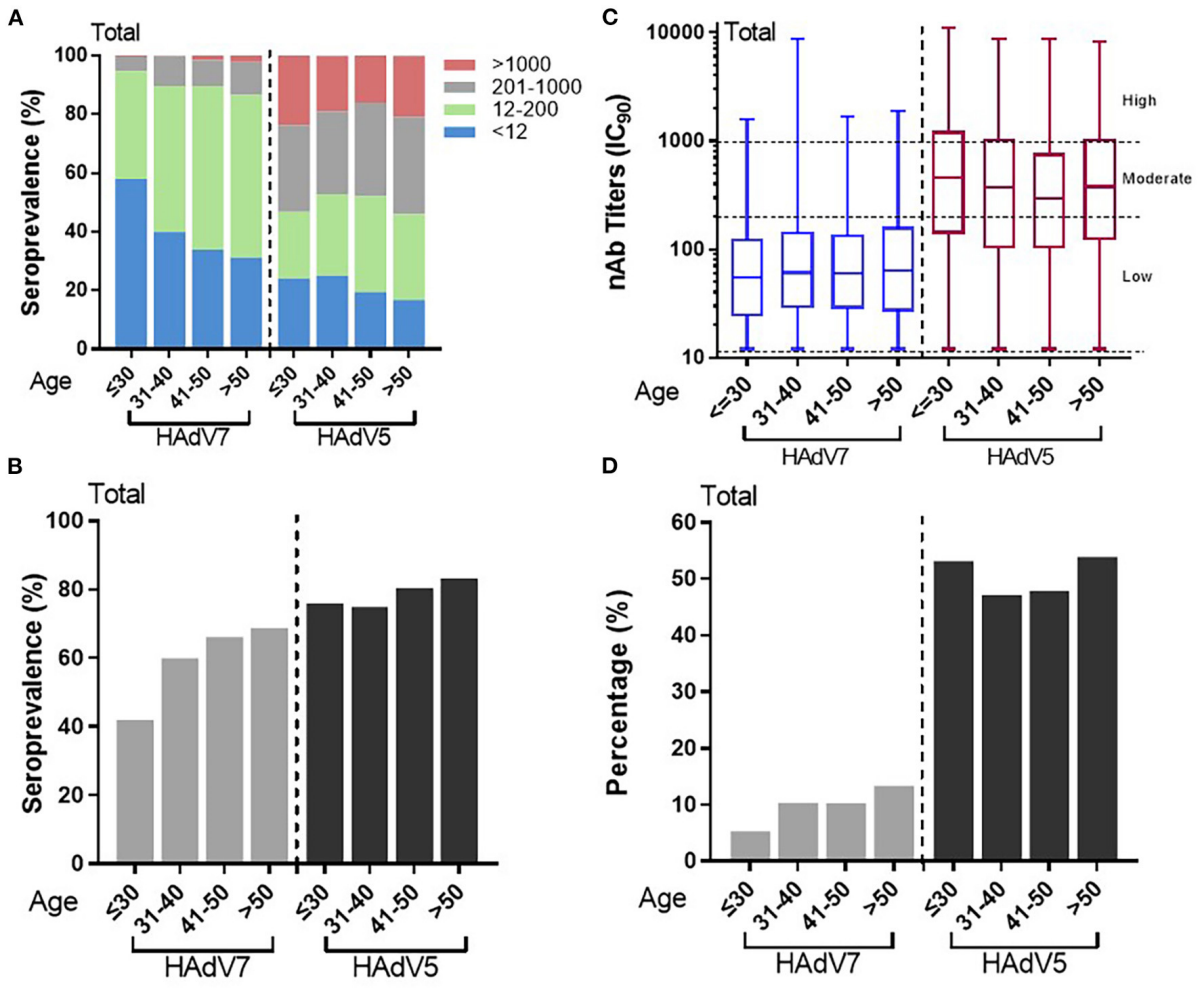
Distributions of HAdV7 and HAdV5 nAbs in the different age groups. **(A)** The nAb distributions in the different age groups are shown. The seropositive rates **(B)** and percentages of serum samples with moderate or high levels of nAbs **(D)** in different age groups are shown. The trends were analyzed by the Cochran–Armitage trend test. **(C)** The titers of HAdV7 and HAdV5 nAbs in seropositive donor age groups are shown. The data were analyzed by the Jonckheere–Terpstra test.

To illustrate the impact of age on HAdV7 nAb seroprevalence, serum nAb levels were stratified into four age groups, as shown in in Figure 4. We analyzed 1,664 serum samples according to age and sex because the remainder of the data were incomplete. The overall HAdV7 seropositive rates were 42.0% (95% CI: 38.0–46.0%), 60.0% (95% CI: 55.5–64.6%), 66.1% (95% CI: 61.3–71.0%), and 68.8% (95% CI: 63.2–74.3%) in the 18–30, 31–40, 41–50, and >50 years age groups, respectively. The seroprevalence significantly increased with age (Cochran–Armitage trend test, *p* < 0.001) (Figures 4A, B), although HAdV7 nAb levels were consistent among the different age groups (Jonckheere-Terpstra test, *p* = 0.093) (Figure 4C). The same trend was also observed among samples from Sierra Leone (Supplementary Figures S2A, B) and China (Supplementary Figures S2C, D). In addition, the proportions of individuals with moderate to high anti-HAdV5 antibodies (titers >200) were 5.3% (95% CI: 3.5–7.1%), 10.4% (95% CI: 7.5–13.2%), 10.3% (95% CI: 7.2–13.4%), and 13.4% (95% CI: 9.3–17.5%) in the corresponding age groups, which also increased with increasing age (Cochran–Armitage trend test, *p* < 0.001) (Figure 4D). In contrast, for HAdV5, a slightly different trend was observed; although increasing age was associated with a higher seroprevalence rate, younger age (< 30 years) appeared to be inversely associated with a higher titer (Figures 4C, D). These results indicated the correlation between age and naturally occurring nAbs against HAdV7, indicating a continuously increasing infection rate of HAdV7 among healthy adults with increasing age.

### Sex independence of HAdV7 nAbs

**Figure 5:**
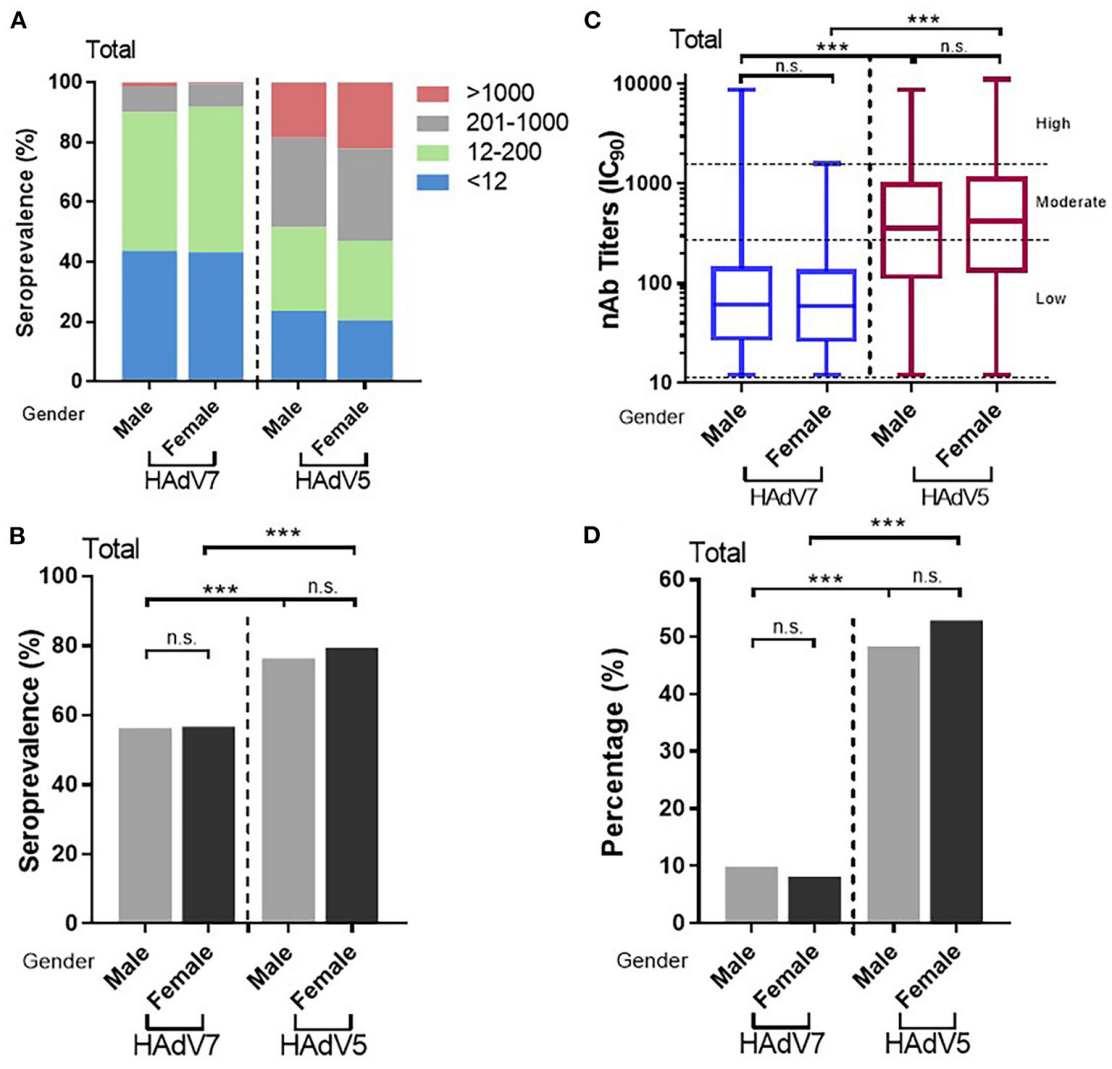
Distributions of HAdV7 and HAdV5 nAbs between sexes. **(A)** The nAb distributions are shown. The seropositive rates **(B)** and percentages of serum samples with moderate or high levels of nAbs **(D)** between sexes were analyzed by the Pearson chi-square test. **(C)** The titers of HAdV7 and HAdV5 nAbs in seropositive individuals by sex are shown and were analyzed by the Mann–Whitney test. n.s., nonsignificant, *p* > 0.05; *p, between 0.01 and 0.05; **p, between 0.01 and 0.001; ***p < 0.001.

As with HAdV5, no statistically significant difference in anti-HAdV7 nAb levels between males and females was observed. The overall HAdV7 seropositive rates in males and females were 56.3% (95% CI: 52.9–59.6%) and 54.7% (95% CI: 51.3–58.1%), respectively, showing no significant differences (Pearson chi-square test) (Figures 5A, B), similar to those observed individually in China and in Sierra Leone (Supplementary Figures S3A, B). The overall HAdV7 nAb titers were also comparable between males and females (Supplementary Figures 5C, D), as well as in Sierra Leone (Supplementary Figure S3C), whereas higher HAdV7 nAb titers were detected in males than in females in China (Mann–Whitney test) (Supplementary Figure S3D). These results demonstrated the indiscriminate infection of HAdV7 between genders.

### Association between HAdV-5 and HAdV-7 infection

**Figure 6:**
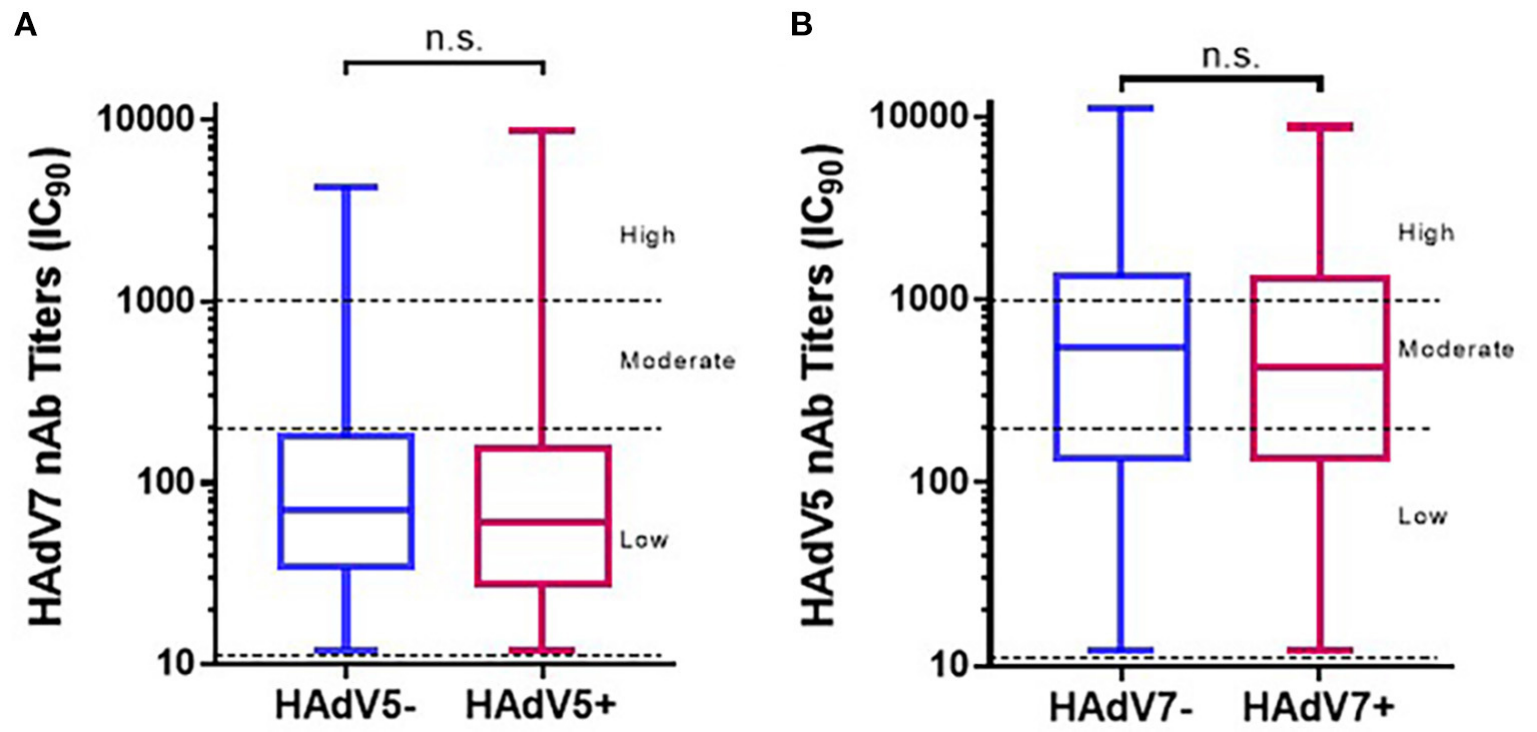
The distributions of HAdV7 and HAdV5 nAbs in single- and double-positive blood donors. The nAb levels of HAdV7 **(A)** and HAdV5 **(B)** in single- and double-positive blood donors are shown. The comparison was analyzed by the Mann–Whitney test. n.s., non-significant.

As shown in Table 1, the HAdV7 seroprevalence among HAdV5-seropositive samples was 59.1% (95% CI: 56.8–61.4%), with no significant difference from that among HAdV5-seronegative samples (57.2%; 95% CI: 53.2–61.2%) (Pearson chi-square test). Likewise, HAdV5 seroprevalence among HAdV7-seropositive samples (75.3%; 95% CI: 73.0–77.6%) was similar to that among HAdV7-seronegative samples (73.9%; 95% CI: 71.1–76.7%) (Pearson chi-square test). In addition, HAdV7 nAb titers in HAdV5-seropositive samples were comparable to those in HAdV5-seronegative samples, and HAdV5 nAb titers were unaffected by HAdV7 seroprevalence (Mann–Whitney test) (Figures 6A, B). These results demonstrated that antibodies against HAdV7 clearly not participated in effective barrier to infections against HAdV5, so as to antibodies against HAdV7 upon HAdV5 infection. Drawing on previous research (24, 27), these data indicated that antibodies against HAdV7 and HAdV5 provided hardly any cross protection.

**Table 1:** Cross-neutralization reactivity between HAdV7 and HAdV5 nAbs.

	**HAdV7 seropositive^a^**	**HAdV7 seronegative^b^**	**HAdV7 seroprevalence^c^ (%)**
HAdV5 seropositive^a^	1,037	719	1,037/1,756 (59.1)
HAdV5 seronegative^b^	340	254	340/594 (57.2)
HAdV-5 seroprevalence (%)^d^	1,037/1,477 (75.3)	719/973 (73.9)	

a The number of HAdV7- or HAdV5-positive serum samples.

b The number of HAdV7- or HAdV5-negative serum samples.

c The rate of HAdV7-positive results in HAdV5-positive or HAdV5-negative serum samples.

d The rate of HAdV5-positive results in HAdV7-negative or HAdV7-positive serum samples.

Correlations were analyzed by the chi-square test, *p* = 0.437.

## Discussion

HAdV7 has been the primary pathogenic adenovirus circulating in China in recent years. The earliest HAdV7 epidemiological investigation was performed in the early 1990's. However, no new reports on HAdV7 nAbs have been published since 2000, probably due to the limitations of testing methods. Herein, we established a simple and quick HAdV7-Luc-based neutralization assay with high selectivity and accuracy, which can be completed within 24 h. This method overcomes the restrictions of traditional tests and provides a novel approach for the study of the seroepidemiology of HAdV7.

In this study, thorough serological surveys of nAbs against HAdV7 were carried out in China and in Sierra Leone. The prevalence of naturally occurring nAbs against HAdV7 varied among populations. Approximately 60% of the Chinese population had HAdV7 nAbs as a result of natural infection earlier in life, based on the few available epidemiologic studies, whereas higher frequencies but lower nAb titers were found in the population in Sierra Leone (Figure 3). Compared with the findings of a survey conducted 20 years ago, the HAdV7 seroprevalence increased from 10–30% (28) to nearly 60%, showing that the rate of infection of HAdV7 increased dramatically. Of interest, we noticed that participants in Xizang, Xinjiang, Beijing and Gansu had higher titers than those in Jiangsu, Sichuan and Hubei (Supplementary Figure S1). Possible reasons for this geographic variation include differences in sanitation and climate as well as regional levels of population immunity. Simultaneously, HAdV7 nAbs were more common in elderly individuals despite all age groups could be affected with no sex specificity (Figures 4, 5); an increased risk of exposure to HAdV7 over time, viral persistence and long-existing ability of nAbs may contribute to this age-specific distribution.

In all the regions, naturally occurring nAbs against HAdV7 had a lower prevalence rate and lower titers than nAbs against HAdV5, which belongs to the HAdV-C subgroup. However, the seroprevalence of HAdV7 was significantly higher than those of HAdV14 (24.8%) and HAdV55 (22.4%) in the same HAdV-B subgroup. In addition, HAdV14 and HAdV55 nAb titers were concentrated at moderate to high levels (>200) (27), while HAdV7 nAb titers were concentrated present at low levels (12–200), confirming that HAdV7 represents a more susceptible type among all HAdV-B family. The high prevalence of HAdV7 infection indicated that ample attention should be paid to the prevention and control of HAdV7 transmission and that regular vaccination could provide protection against HAdV7-related diseases.

HAdV vectors, especially comparatively developed HAdV5 vectors, have been widely used for protein delivery in practices such as gene therapy, cancer virotherapy and vaccine development. There are numerous advantages to using adenoviruses as vectors, including (1) the capacity to infect a wide variety of dividing and non-dividing cells; (2) the feasibility of oral or intranasal administration; (3) excellent efficiency in accommodating large transgenes; (4) a low frequency of genomic integration; and (5) the relative ease of laboratorial construction of recombinant virus (29, 30). However, preexisting immunity against HAdV has a negative impact on efficient gene delivery, and research is currently being performed to identify potential options to overcome preexisting HAdV5 immunity. A previous epidemiological study showed that the overall proportions of anti-HAdV5-, anti-HAdV6-, anti-HAdV26- and anti-HAdV36-seropositive individuals were 85.2, 68.5, 58.0, and 46.4%, respectively (31). Herein, the detected prevalence of HAdV7 was 58.6%, especially with 47.0% remained at low levels. Because low levels of nAbs have shown no negative effect on the protective efficacy of HAdV-vector-based vaccines (32), these differences support the possibility that recombinant HAdV7 vectors could be used as alternatives to HAdV5 vectors in vaccine development.

Antibody cross-reactivity between HAdV7 and other adenoviruses in people is suspected but has not been demonstrated to date. Our results showed that there was barely any cross-reactivity between nAbs against adenovirus types 5 and 7 (Figure 6), which was consistent with the relatively high frequency of adenoviral coinfections with HAdV-B and HAdV-C (33). Based on these observations, it seems that the combination of different adenoviruses for heterologous priming or chimeric vector construction could be a promising and attractive research focus in the future.

There are limitations in this study that need to be improved. For example, the nAb titers are highly variable in overall supposedly owing to interpersonal variability and the conclusions would be more precise if larger sample sizes were included in each region.

In summary, the seroepidemiological assessment based on the HAdV7-Luc recombinant virus revealed that HAdV7 circulation was prevalent among populations in China and in Africa. The elucidation of geographic variations in preexisting immunity to HAdV7 highlights the importance of HAdV7 surveillance and may pave the way for the clinical application of HAdV7-based vaccines and gene therapy tools.

## Materials and methods

### Establishment of a reporter-expressing recombinant HAdV7-Luc virus

The 5' untranslated region (UTR) and 3' UTR of HAdV7 were polymerase chain reaction (PCR)-amplified from the HAdV7-GZ08 (GQ478341) strain using the primers Ad7-re-Left-F/R and Ad7-re-Right-F/R, respectively, and the kanamycin coding sequence and PBR322 replication origin site from our previously generated pAd4-dE3-luc plasmid were amplified using primers Ad7-re-V1-F/R. Then, a UTR_kanamycin_ PBR322_UTR shuttle plasmid was produced using a Gibson Assembly Cloning Kit and designated pAd7-re. Next, pAd7-V0 was formed during homologous recombination of the HAdV7 genome with pAd7-re in *Escherichia coli* BJ5183 competent cells (Agilent Technologies, CA, USA). A replication-incompetent pAd7ΔE3 mutant was produced using the restriction enzyme EcoRI (New England Biolabs, MA, USA). For the construction of pAd7ΔE3-Luc, we first amplified the luciferase expression cassette from our previously generated pAd4ΔE3-Luc by PCR using the primers Ad7-Luc1-F and Ad7-Luc2-R. The E1 region was spliced from pAd7ΔE3 *via* digestion with AatII and BsiWI and replaced with the ligated luciferase amplicon. The origin shuttle plasmid pAd7-re (GenBank accession number: OP902171) and the ultimately used plasmid pAd7ΔE3-Luc (GenBank accession number: OP902172) were well confirmed by sequencing, demonstrating the correctness of the recombination. The oligonucleotide primers used in this study are summarized in Supplementary Table S1.

To produce recombinant HAdV7-Luc viruses, HEK293 cells were transfected with the linearized pAd7ΔE3-Luc plasmid. Cells were washed with PBS once and refreshed with DMEM the day after transfection. The proliferative viruses were purified by ion-exchange chromatography on a Source 30Q column (GE Healthcare, Beijing, China). Then, HAdV7 viral titers were determined in HEK293 cells using an Adeno-XTM Rapid Titer Kit (Clontech, CA, USA), as previously described (24). In brief, HEK293 cells [5 × 10 (4) cells/mL] in 24-well plates were infected with HAdV7-Luc after 10-fold serial dilution. Specific anti-adenovirus hexon antibodies were used to label the infected viruses, and the visualized infected cells were quantified using a microscope. The infectious units (IFU)/mL was calculated as [infected cells/field) × (field/well)/(volume virus (mL) × (dilution factor)].

### Blood samples

A total of 2,350 serum samples from individuals in China and Sierra Leone in West Africa were included in this seroepidemiological study. Among them, 734 samples were from Hubei obtained in March 2020, 43 from Gansu obtained in August 2019, 169 from Sichuan obtained in August 2019, 273 from Beijing obtained in March 2017, 120 from Jiangsu obtained throughout the year 2015, 206 from Xinjiang obtained in August 2018, 307 from Xizang obtained in August 2020, and 498 from Freetown, the capital of Sierra Leone, obtained in October 2015. The blood donors were all healthy adults between 18 and 65 years old. A total of 1,664 serum samples had complete age and sex information, while the rest of the samples had incomplete data. Samples collected in Sierra Leone (34), Hubei (35–37), and Jiangsu (38) were involved in the clinical trial of an HAdV-5-based vaccine. The demographic data of the enrolled participants are summarized in Table 2, and no statistically significant differences were identified with regard to sex and age. The sera obtained after centrifugation at 2,000 rpm for 15 min were kept in a deep freezer at −20°C and inactivated for 30 min at 56°C before use.

**Table 2:** Demographics of all the serum sample donors (*n* = 2,350).

	**Hubei**	**Gansu**	**Sichuan**	**Beijing**	**Jiangsu**	**Xinjiang**	**Freetown**	**Xizang**	**Overall**
Regional samples, *n* (%)	734 (100)	43 (100)	169 (100)	273 (100)	120 (100)	206 (100)	498 (100)	307 (100)	2,350 (100)
**Age group, years;** ***n*** **(%)**									
18–30	245 (33.38)	-	-	53 (19.41)	14 (11.67)	-	244 (49.00)	32 (10.42)	588 (25.02)
31–40	177 (24.11)	-	-	72 (26.37)	31 (25.83)	-	146 (29.32)	20 (6.51)	446 (18.98)
41–50	150 (20.44)	-	-	68 (24.91)	43 (35.83)	-	108 (21.69)	5 (1.63)	374 (15.91)
>50	162 (22.07)	-	-	80 (29.30)	32 (26.67)	-	0	0	274 (11.66)
Unknown^a^	0	43 (100)	169 (100)	0	0	206 (100)	0	250 (81.43)	668 (28.43)
**Sex**, ***n*** **(%)**									
Male	368 (50.14)	-	-	120 (43.96)	59 (49.17)	-	268 (53.82)	47 (15.31)	862 (36.68)
Female	366 (49.86)	-	-	135 (49.45)	61 (50.83)	-	230 (46.18)	10 (3.26)	802 (34.13)
Unknown^b^	0	43 (100)	169 (100)	18 (6.59)	0	206 (100)	0	250 (81.43)	686 (29.19)

a Age information of 668 people was unknown.

b Sex information of 686 people was unknown.

### Ethical approval

The study protocol was approved by the Scientific Review Committee and the relevant institutional review boards. The samples collected in Sierra Leone were involved in the clinical trial of a HAdV5-based vaccine, which was approved by the Sierra Leone Ethics Committee and the Pharmacy Board of Sierra Leone and was performed in accordance with the Declaration of Helsinki.

### Neutralization assay based on the HAdV7-Luc reporter virus

The adenovirus neutralization assay was performed as previously described (24), except a newly synthesized HAdV7-Luc virus was used. In brief, HAdV7-Luc was incubated with 3-fold serial dilutions of heat-inactivated sera (starting at 1:12 and ending at 1:8,748) for 1 h at 37°C before 2 × 10 (4) A549 cells were added to 96-well plates. Simultaneously, diluent without serum was used as a negative control. After incubation for 24 h at 37°C, the inoculum was removed, and luciferase expression was detected using a Luciferase Assay System (Promega, Madison, WI). The reciprocal of the dilution multiple corresponding to a 90% reduction in the fluorescence value (IC_90_) compared to that of the negative control was designated as the nAb titer, using non-linear regression with four parameters in GraphPad.

In addition, a traditional cytopathic effect (CPE) assay was conducted to validate the stability, reliability, and accuracy of the HAdV7-Luc-based neutralization assay. A mixture of HAdV7-Luc and serum was incubated with HEK293 cells for 6 days before cell viability measurement using a Cell Proliferation Assay kit (Promega, Madison, WI). The correlation of these two methods was then analyzed using Spearman correlation analysis.

### Neutralization assay based on the HAdV5-Luc reporter virus

The recombinant HAdV-5 vector-based luciferase expressing virus was constructed using the AdMax system (Microbix Biosystems, ON, Canada) as detailedly described previously (24, 39). Briefly, E1/E3 region of Ad5-based vector was substituted by a luciferase expression cassette, then the recombinant adenovirus was generated in the adenoviral packaging cells and propagated on HEK293 cells. HAdV-5-specific nAbs were assessed with the same method described above instead that HAdV5-Luc reporter virus was used.

### Statistical analysis

Descriptive statistics were generated. The software PASW Statistics 18 and GraphPad Prism 7.0 were used for data analysis. Seropositivity ratios and nAb titers were evaluated using the Pearson chi-square test and Mann–Whitney test, respectively. The trend of the association of seroprevalence with age was analyzed by the Cochran–Armitage trend test, and the trends in nAb titers among age groups were analyzed by the Jonckheere–Terpstra test. n.s., nonsignificant, *p* > 0.05; ^*^p, between 0.01 and 0.05; ^**^p, between 0.01 and 0.001; ^***^p < 0.001.

## Data availability statement

The raw data supporting the conclusions of this article will be made available by the authors, without undue reservation.

## Ethics statement

The studies involving human participants were reviewed and approved by the Institutional Review Board of the Jiangsu Provincial Center of Disease Control and Prevention, the Medical Ethics Committee of the Academy of Military Medical Sciences, and the Sierra Leone Ethics and Scientific Review Committee. The patients/participants provided their written informed consent to participate in this study.

## Author contributions

Conceptualization: LH and WC. Investigation: BW, JL, SW, YW, YC, YZ, XS, ZZhao, ZZhan, JZ, and RY. Statistical analysis: BW and YZ. Writing—original draft: BW. Writing—review and editing: BW and LH. All authors have read and agreed to the published version of the manuscript.
