# Supplementary material for: A seroepidemiological survey of adenovirus type 7 circulation among healthy adults in China and in Sierra Leone, West Africa
Source: Front Public Health. 2023 Feb 6;11:1095343. doi: 10.3389/fpubh.2023.1095343 (PMC9940762; doi:10.3389/fpubh.2023.1095343)
Supplement: Supplementary file 1 [file Data_Sheet_1.pdf]

Supplementary Materials for

A seroepidemiological survey of adenovirus type 7 circulation among healthy adults in China and in Sierra Leone, West Africa.

Busen Wang[1,#], Jianhua Li[2,#], Shipo Wu[1], Yudong Wang[1], Yi Chen[1], Yanfang Zhai[1], Xiaohong Song[1], Zhenghao Zhao[1], Zhe Zhang[1], Jinlong Zhang[1], Rui Yu[1], Lihua Hou[1,*], Wei Chen[1,*]

[1] Beijing Institute of Biotechnology, No. 20 Dongdajie Street, Fengtai District, Beijing 100071, China.
[2]Zhejiang Provincial Center of Disease Control and Prevention

[#]These authors contributed equally to this work.

*Correspondence to: houlihua@sina.com (L.H), cw0226@foxmail.com (W.C)

This PDF file includes: Figures S1 to S3 and Table S1.

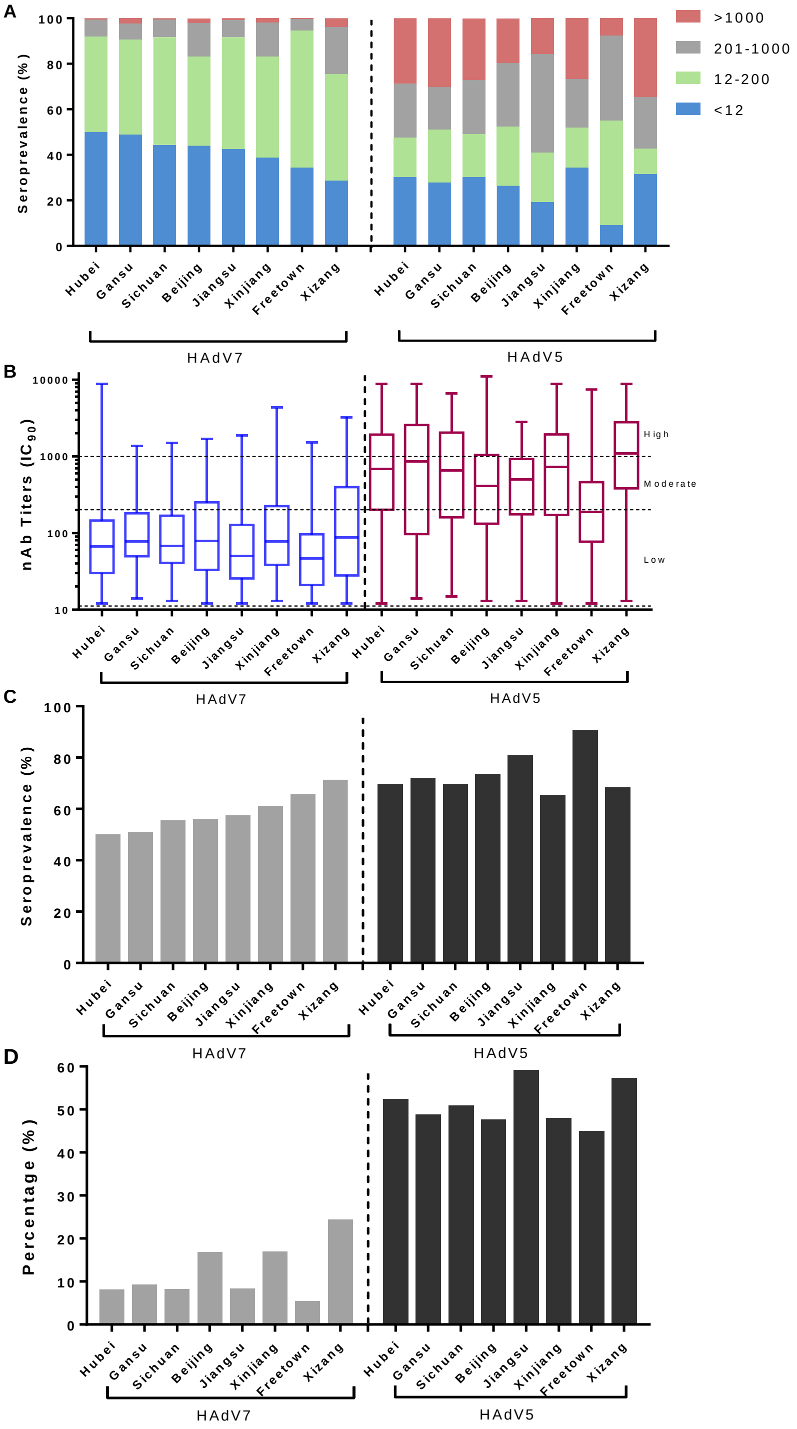

Supplementary Figure S1 Seroprevalences of HAdV7 and HAdV5 nAbs in different regions. (A) The nAb distributions are shown. (B) The nAb titers in HAdV7- and HAdV5-positive serum samples. (C) The seropositive rates in different regions. (D) The percentages of serum samples with moderate or high levels of nAbs.

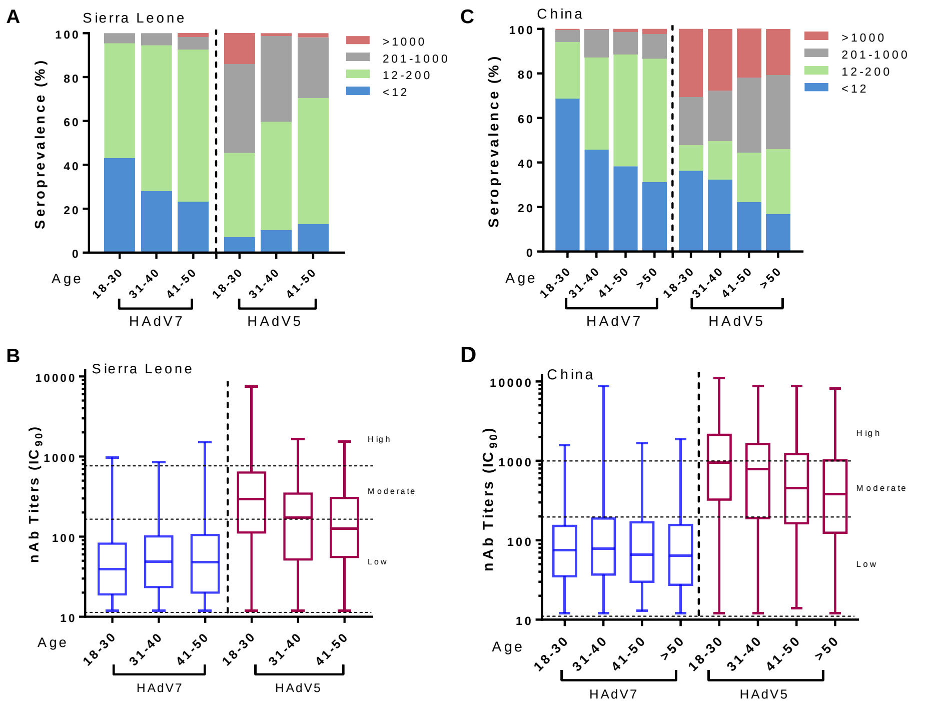

Supplementary Figure S2 Distributions of HAdV7 and HAdV5 nAbs in different age groups in China and Sierra Leone. (A and C) The nAb distributions in different age groups in China and Sierra Leone. (B and D) The titers of HAdV7 and HAdV5 nAbs in seropositive donor age groups in China and Sierra Leone. The data were analyzed by the Jonckheere–Terpstra test.

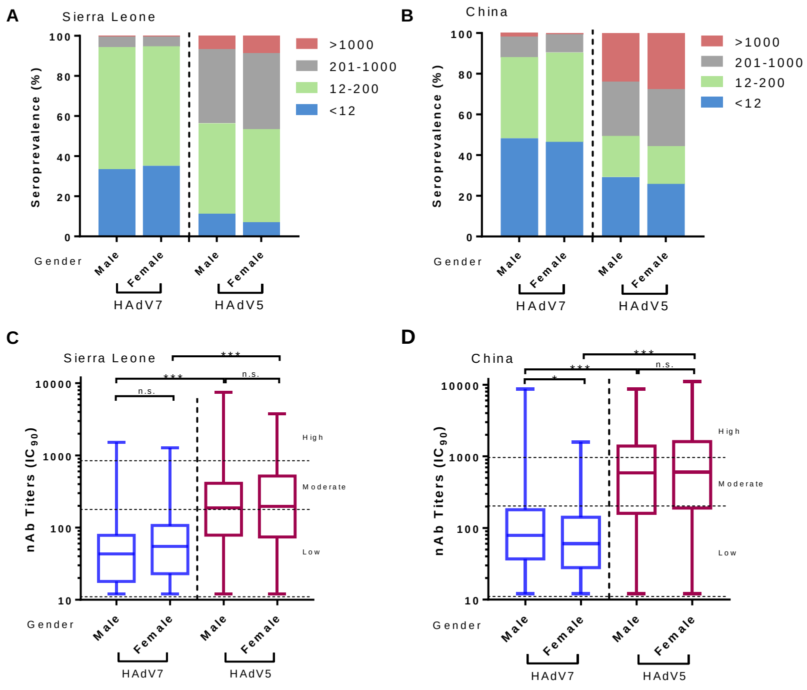

Supplementary Figure S3 Distributions of HAdV7 and HAdV5 nAbs between sexes in China and Sierra Leone. (A-B) The nAb distributions between sexes in Sierra Leone (A) and China (B). (C-D) The titers of HAdV7 and HAdV5 nAbs in seropositive donors by sex in Sierra Leone (C) and China (D), analyzed by the Mann–Whitney test. n.s., nonsignificant, *p* > 0.05; **p*, between 0.01 and 0.05; ** *p*, between 0.01 and 0.001; ****p* < 0.001.

Supplementary Table S1

Oligonucleotide primers used for construction of the HAdV7-Luc recombinant plasmid.

Primers	Sequences (5' to 3')
Ad7-re-Left-F	CTATCTATATAATATACCTTATAGATGG
Ad7-re-Left-R	CAAGGGCTCCAGTTTGTTTAAACCCAGTGCAGGCAAGCACCAAATG
Ad7-re-Right-F	AAACTGGAGCCCTTGTCACTGC
Ad7-re-Right-R	GGAACATAATGGTGCGCGATCGCCTATCTATATAATATACCTTATAGATGG
Ad7-re-V1-F	GCACCATTATGTTCCGGATCTGC
Ad7-re-V1-R	TACGCGGTCGCTGTTAAAAGG
Ad7-Luc1-F	CTGATAGGATATGAGG
Ad7-Luc2-R	GAGACTCCGTCCGAGGCG
